# Biochemical profile of human infant cerebrospinal fluid in intraventricular hemorrhage and post-hemorrhagic hydrocephalus of prematurity

**DOI:** 10.1186/s12987-021-00295-8

**Published:** 2021-12-24

**Authors:** Ayodamola Otun, Diego M. Morales, Maria Garcia-Bonilla, Seth Goldberg, Leandro Castaneyra-Ruiz, Yan Yan, Albert M. Isaacs, Jennifer M. Strahle, James P. McAllister, David D. Limbrick

**Affiliations:** 1 Department of Neurosurgery, Washington University in St. Louis School of Medicine, St. Louis, MO 63110 USA; 2 Department of Nephrology, Washington University in St. Louis School of Medicine, St. Louis, MO 63110 USA; 3 Department of Surgery, Washington University in St. Louis School of Medicine, St. Louis, MO 63110 USA; 4 Division of Neurosurgery, Department of Clinical Neurosciences, University of Calgary, Calgary, AB T2N 2T9 Canada; 5 Children’s Hospital Orange County, Children’s Research Institute, Orange, CA 92868 USA

**Keywords:** Post-hemorrhagic hydrocephalus (PHH), Intraventricular hemorrhage (IVH), CSF osmolality, CSF electrolytes, Cerebrospinal fluid (CSF)

## Abstract

**Background:**

Intraventricular hemorrhage (IVH) and post-hemorrhagic hydrocephalus (PHH) have a complex pathophysiology involving inflammatory response, ventricular zone and cell–cell junction disruption, and choroid-plexus (ChP) hypersecretion. Increased cerebrospinal fluid (CSF) cytokines, extracellular matrix proteins, and blood metabolites have been noted in IVH/PHH, but osmolality and electrolyte disturbances have not been evaluated in human infants with these conditions. We hypothesized that CSF total protein, osmolality, electrolytes, and immune cells increase in PHH.

**Methods:**

CSF samples were obtained from lumbar punctures of control infants and infants with IVH prior to the development of PHH and any neurosurgical intervention. Osmolality, total protein, and electrolytes were measured in 52 infants (18 controls, 10 low grade (LG) IVH, 13 high grade (HG) IVH, and 11 PHH). Serum electrolyte concentrations, and CSF and serum cell counts within 1-day of clinical sampling were obtained from clinical charts. Frontal occipital horn ratio (FOR) was measured for estimating the degree of ventriculomegaly. Dunn or Tukey’s post-test ANOVA analysis were used for pair-wise comparisons.

**Results:**

CSF osmolality, sodium, potassium, and chloride were elevated in PHH compared to control (p = 0.012 − < 0.0001), LGIVH (p = 0.023 − < 0.0001), and HGIVH (p = 0.015 − 0.0003), while magnesium and calcium levels were higher compared to control (p = 0.031) and LGIVH (p = 0.041). CSF total protein was higher in both HGIVH and PHH compared to control (p = 0.0009 and 0.0006 respectively) and LGIVH (p = 0.034 and 0.028 respectively). These differences were not reflected in serum electrolyte concentrations nor calculated osmolality across the groups. However, quantitatively, CSF sodium and chloride contributed 86% of CSF osmolality change between control and PHH; and CSF osmolality positively correlated with CSF sodium (r, p = 0.55,0.0015), potassium (r, p = 0.51,0.0041), chloride (r, p = 0.60,0.0004), but not total protein across the entire patient cohort. CSF total cells (p = 0.012), total nucleated cells (p = 0.0005), and percent monocyte (p = 0.016) were elevated in PHH compared to control. Serum white blood cell count increased in PHH compared to control (p = 0.042) but there were no differences in serum cell differential across groups. CSF total nucleated cells also positively correlated with CSF osmolality, sodium, potassium, and total protein (p = 0.025 − 0.0008) in the whole cohort.

**Conclusions:**

CSF osmolality increased in PHH, largely driven by electrolyte changes rather than protein levels. However, serum electrolytes levels were unchanged across groups. CSF osmolality and electrolyte changes were correlated with CSF total nucleated cells which were also increased in PHH, further suggesting PHH is a neuro-inflammatory condition.

**Supplementary Information:**

The online version contains supplementary material available at 10.1186/s12987-021-00295-8.

## Introduction

High grade intraventricular hemorrhage (HGIVH), defined as IVH with blood filling more than 50% of the ventricular volume (Papile grades III–IV) [1], affects nearly 20% of preterm infants with post-menstrual age < 28 weeks [2, 3] and is associated with post-hemorrhagic hydrocephalus (PHH) in 25–50% of cases [3, 4]. HGIVH carries a risk of substantial neurological morbidity, but outcomes in PHH are among the worst in newborn medicine, with up to 85% of infants developing neurodevelopmental delay, visual impairment, cerebral palsy, deafness, or some combination of these [3, 5–7]. The pathophysiology of disability in HGIVH and PHH remain to be elucidated [8, 9].

PHH is a complex condition that involves neuro-inflammation [8], alterations in ventricular zone (VZ) junctional biology [10–14], and choroid plexus (ChP) hypersecretion [15], among other processes. CSF studies of human infants with PHH [2, 4, 16, 17] have shown elevated inflammatory markers (chemokines and cytokines) [18–24]; mediators of neurodevelopment [25], extracellular matrix proteins [2, 7, 25, 26]; blood-associated proteins and metabolites (hemoglobin, ferritin)[6, 27–29]; proteins involved in coagulation (fibrin and fibrin degradation products)[30, 31]; and cell junction proteins [2, 7]. However, only a limited number of studies have examined the biochemical profile of CSF in other forms of hydrocephalus [32, 33], and no studies have reported these in PHH in human preterm infants to date. CSF osmolality and electrolyte concentration changes have been shown in diabetes ketoacidosis, febrile children, and a constellation of neurological diagnoses such as polyneuropathy, multiple sclerosis, amyotrophic lateral sclerosis, etc. [34–37]. Notably, CSF studies from animal models of hydrocephalus have implicated CSF osmolality in the development of ventriculomegaly [38, 39].

We hypothesized that PHH in human preterm infants is associated with alterations in the biochemical profile of CSF, including CSF electrolytes, total protein, and osmolality. To examine this hypothesis, we measured each of CSF osmolality, protein levels, and electrolyte concentrations in lumbar CSF and serum obtained from human infants with no known neurological injury, IVH (grades I/II and III/IV), and PHH. PHH was associated with increased CSF protein, osmolality, and specific electrolytes likely to impact osmotic and oncotic pressure. These findings provide novel insights into the putative pathophysiology of ventriculomegaly in PHH. To our knowledge, this is the first study quantifying the biochemical profile of CSF in human infants with PHH.

## Materials and methods

### Research subjects and study design

We retrospectively analyzed human CSF prospectively collected at Washington University/St. Louis Children’s Hospital between November 2009 and December 2017. Included were infants born at post-menstrual age (PMA) < 34 weeks with birth weight < 2000 g who underwent lumbar puncture (LP) in the St. Louis Children’s Hospital (SLCH) Neonatal Intensive Care Unit (NICU) as a part of routine care. Neonates with no known neurologic disease who underwent a LP for sepsis evaluation where the evaluation and all cultures were negative were included as controls. Papile grading scale [1, 40] was used by a neuroradiologist to assign IVH grade. Neonates diagnosed with IVH grade I and II were included as low grade IVH (LGIVH) and IVH III-IV as high grade IVH (HGIVH). PHH was defined based on the Hydrocephalus Clinical Research Network (HCRN) guidelines[41] including infants with frontal occipital horn ratio (FOR) ≥ 0.55 who required surgical treatment. PHH determination was also confirmed by an attending pediatric neurosurgeon (D.D.L., J.M.S.).

All CSF samples were acquired via standard LP and serum samples were acquired via routine clinical venipuncture PHH patients underwent temporizing neurosurgical treatment with a ventricular reservoir followed by permanent treatment with a CSF shunt, or, in some cases, endoscopic third ventriculostomy with choroid plexus cauterization. LP in IVH and PHH group was performed after the occurrence of IVH but prior to PHH diagnosis and any neurosurgical intervention.

All protocols and procedures were approved by the Washington University Human Research Protection Office and Institutional Review Board with a waiver of written informed consent (HRPO# 201203126).

### Comorbidities

The general health status of the neonates was assessed with the clinical risk index for babies minus temperature (CRIB II – T) [42] and a modified version of complex chronic conditions (CCC) [43]. The CRIB II-T score is a validated score that assesses initial mortality risk and illness severity within 1 h of admission [44, 45]. It uses the birth weight, sex, gestational age, and base excess to provide a score ranging from 1 to 27. The CCC is a list of chronic conditions relevant to the pediatric population organized into categories. The modified CCC which redefines the neuromuscular category to exclude hydrocephalus has been used in many studies of hydrocephalus [46–49]. We also reported neonatal abstinence syndrome (NAS) and infection (chorioamnionitis, n = 2; congenital syphilis, n = 1; osteomyelitis, n = 1) to describe the current comorbidities particularly relevant to the cohort of patients included in this study more robustly.

### Specimen collection and processing

CSF samples were collected by the NICU clinical team at SLCH using standard, sterile procedures for LP. CSF samples were sent to the SLCH clinical laboratory for clinically indicated microbiological culture (5-day sutveillance) and laboratory evaluation. Excess CSF or “saved” CSF samples were centrifuged at 1000 g for six minutes and the supernatant aliquoted, frozen -20 °C for 3 months before transfer to the Washington University Neonatal CSF Repository for freezing and storage at − 80 °C until the time of experimental analysis, as described previously [26, 50]. CSF cell count, and serum electrolyte, cell count, and differential analysis were obtained from the electronic medical record. Only serum samples within 1 day of the CSF sample collection were included in the study analysis.

### Osmolality, protein, and electrolyte measurements

Osmolality was measured using the VAPRO vapor pressure osmometer (Wescor 5520), as described previously for analysis of CSF and other biofluids [51–53]. The osmometer was stored in a dry room with stable temperature. The instrument was calibrated prior to each use with a standard solution of 290 mmol/L, with an error range of ± 3 mmol/L. Following the manufacturer’s recommendation based on expected variation, two readings were obtained for each sample, with a third obtained if the difference between the two initial readings was > 5 mmol/L. The mean of the readings was recorded and used in the analysis. All samples were stored at − 80 °C before use and at 0 °C in between readings.

CSF electrolyte (sodium, potassium, chloride, bicarbonate, glucose, magnesium, and calcium) measurements were performed in the Washington University core clinical laboratory for clinical studies after instrument validation using artificial CSF (10 mM glucose, 3.0 mM KCl, 1.2 mM KH2PO4, 2 mM MgCl2, 2.6 mM CaCl2, 124 mM NaCl, and 26 mM NaHCO3, see Additional file 1 for full description) to ensure reproducibility, linearity, and accuracy. All electrolyte assays were performed on a Roche Cobas c501 module of a Roche Cobas 6000 platform. Sodium, potassium, and chloride assays utilized an ion-selective electrode, and their concentrations were determined by the Nernst equation. Magnesium assay was based on a colorimetric endpoint using reaction with xylidyl blue, while calcium assay was based on a complex of 5-nitro-5’-methyl-5-(1,2-bis(0-aminophenoxy)ethan-N,N,N’N’-tetraacetic acid. Glucose was measured using the hexokinase coupled-enzyme assay method, while bicarbonate measurement was based on a phosphoenolpyruvate carboxylase/malate dehydrogenase coupled enzyme assay. Total protein was measured using the Pierce Bicinchoninic Acid protein assay kit (Thermo-scientific). The assay was run following the manufacturer’s manual [2].

### Statistical analysis

Mean and standard deviation were calculated for each experimental group for each variable measured. All statistical analysis were performed on GraphPad Prism 9 and statistical significance was set at an alpha level < 0.05. Normality tests (Shapiro–Wilk) were performed and, if the data were normally distributed, ANOVA with Tukey post-test analyses were conducted. Otherwise, Kruskal–Wallis ANOVA with Dunn’s post-test analyses were performed for non-parametric data. Pearson or Spearman correlation test were also performed based on normality test result.

## Results

### Patient characteristics

A total of 52 patients were included in this study: 18 Control, 10 LGIVH, 13 HGIVH, and 11 PHH infants. There was a higher percentage of males in all groups (Table 1). There was no statistically significant difference between the PMA of the PHH subjects (27.6 ± 3.1 weeks) compared to control (PMA 31.1 ± 2.9; p = 0.068), LGIVH (PMA 28.2 ± 2.8; p > 0.99), or HGIVH (PMA 26.2 ± 2.7; p > 0.99). However, HGIVH infants were younger than control (p = 0.0005). Crib II – T score was similar between PHH (8.7 ± 4.2) and control (CRIB II-T score 4.6 ± 4; p = 0.15) but higher in HGIVH (CRIB II-T score 10.7 ± 3.9) compared to control (p = 0.0076). The CCC score was similar across the groups (p = 0.39). The most common category of comorbid condition in the entire cohort was respiratory followed by cardiovascular. FOR was higher in PHH compared to control (p < 0.0001), LGIVH (p < 0.0001), and HGIVH (p < 0.0001). FOR was also higher in HGIVH compared to LGIVH (p = 0.034) and control (p = 0.0003).

**Table 1:** Summary of patient characteristics

	Control (N = 18)	LGIVH (N = 10)	HGIVH (N = 13)	PHH (N = 11)	P value
Sex					
Male	13 (72.2)	6 (60.0)	12 (92.3)	9 (81.8)	
Female	5 (27.8)	4 (40.0)	1 (7.7)	2 (18.2)	
PMA birth (weeks)	31.1 ± 2.9	28.2 ± 2.8	26.2 ± 2.7	27.6 ± 3.1	0.0009 control vs HGIVH (0.0005)
Birth Weight (g)	1388.0 ± 395.5	1453.0 ± 752.8	986.2 ± 396.2	1041.1 ± 260.9	0.037 control vs HGIVH (0.032)
CRIB II—T Score	4.6 ± 4.5	6.9 ± 4.7	10.7 ± 3.9	8.7 ± 4.2	0.012 control vs HGIVH (0.0076)
Complex Chronic Conditions	0.8 ± 0.8	1.0 ± 0.7	0.8 ± 1.2	1.4 ± 1.1	0.39
None	9 (50.0)	2 (20.0)	7 (53.8)	3 (27.3)	
1–2	9 (50.0)	8 (80.0)	5 (38.5)	6 (54.5)	
3–4	0	0	1 (7.7)	2 (18.2)	
> 4	0	0	0	0	
Respiratory	6	4 (40.0)	4 (30.8)	7 (63.6)	
Cardiovascular	3 (16.7)	0	2 (15.4)	4 (36.4)	
Neuromuscular	2 (11.1)	0	0	0	
Ophthalmologic	1 (5.6)	1 (10.0)	1 (7.7)	0	
Gastrointestinal	0	0	2 (15.4)	1 (9.1)	
Infectious	0	3 (30.0)	0	0	
Hematologic	1 (5.6)	0	0	0	
NAS	1 (5.6)	2 (20.0)	0	0	
FOR measurement	0.35 ± 0.04	0.41 ± 0.07	0.49 ± 0.06	0.65 ± 0.03	< 0.0001 Control vs HGIVH (0.0003) and PHH (< 0.0001); LGIVH vs HGIVH (0.034) and PHH (< 0.0001); HGIVH vs PHH (< 0.0001)

Values are reported as N (column %) or mean ± standard deviation. In the “P value’ column, ANOVA summary p-value was recorded on the top row while significant pairwise post-test P value were recorded on the bottom row. All pairwise comparisons were analyzed based on Dunn’s or Tukey’s post-test ANOVA depending on the distribution of the data (normal distribution or not). N, sample size; PMA, post-menstrual age; NAS, neonatal abstinence syndrome; FOR, frontal-occipital horn ratio

### CSF, serum culture and cell counts

CSF and serum samples from all patients included in this study were cultured for five days and no growth of any micro-organism was noted. Compared to control, CSF total cells (p = 0.012), total nucleated cells (p = 0.0005), and percent monocytes (p = 0.016) were higher in PHH (Table 2). Total nucleated cells (p = 0.0079) and percent lymphocyte (p = 0.043) were elevated in HGIVH compared to control. Comparing LGIVH to HGIVH and PHH, total nucleated cells were also elevated in both HGIVH (p = 0.011) and PHH (p = 0.0010). There were no changes in percent neutrophils (p = 0.24) and macrophages (p = 0.23) in the CSF across all groups. In the serum, on the other hand, only total white blood cell (WBC) count was increased in PHH compared to control (p = 0.042) and no changes in the relative distribution of the different serum white blood cell types. Serum platelet was higher in HGIVH compared to control (p = 0.030), but no other differences were observed across groups.

**Table 2:** Serum and CSF cellularity

	Control (N = 18)	LGIVH (N = 10)	HGIVH (N = 13)	PHH (N = 11)	P value
CSF					
Total cells (cells/mcl)	1711 ± 4305	452.7 ± 596.1	12,572 ± 24,771	39,788 ± 70,835	0.014 Control vs PHH (0.012)
Nucleated cells (cells/mcl)	3.8 ± 4.2	2.8 ± 3.2	67.2 ± 95.4	287.7 ± 433.2	Control vs HGIVH (0.0079) and PHH (0.0005); LGIVH vs HGIVH (0.011) and PHH (0.0010)
Lymphocytes (%)	25.7 ± 16.3	23.0 ± 17.9	10.4 ± 4.5	17.1 ± 11.6	0.051 Control vs HGIVH (0.043)
Neutrophils (%)	25.0 ± 11.7	30.0 ± 30.3	38.2 ± 32.4	49.4 ± 28.7	0.24
Monocytes (%)	48.7 ± 22.9	46.6 ± 34.2	39.6 ± 32.6	16.3 ± 10.7	0.024 Control vs PHH (0.016)
Macrophages (%)	10.2 ± 9.2	29.0 ± 25.2	28.3 ± 38.2	10.2 ± 6.1	0.23
Serum					
WBC (K/cumm)	12.1 ± 6.6	14.8 ± 9.9	15.0 ± 10.0	22.9 ± 10.0	0.051
Control vs PHH (0.042)					
RBC (K/cumm)	3.5 ± 0.7	3.7 ± 0.9	3.2 ± 0.6	3.5 ± 0.4	0.34
Platelet (K/cumm)	329.6 ± 134.9	223.0 ± 66.6	202.9 ± 104.5	251.5 ± 92.2	0.032 Control vs HGIVH (0.030)
Neutrophils (%)	47.5 ± 21.7	46.1 ± 25.1	42.2 ± 21.9	55.0 ± 13.4	0.53
Lymphocytes (%)	40.7 ± 20.0	31.9 ± 18.6	36.5 ± 17.7	27.9 ± 11.8	0.37
Monocytes (%)	8.3 ± 4.2	11.3 ± 4.5	9.5 ± 5.9	12.0 ± 5.4	0.18

Values are reported as N (column %) or mean ± standard deviation. In the “P value’ column, ANOVA summary p-value was recorded on the top row while significant pairwise post-test P value were recorded on the bottom row. All pairwise comparisons were analyzed based on Dunn’s or Tukey’s post-test ANOVA depending on the distribution of the data (normal distribution or not). Cell differential reported as % of total WBC. WBC, white blood cells; RBC, red blood cells

### CSF osmolality, total protein, and glucose

**Fig. 1:**
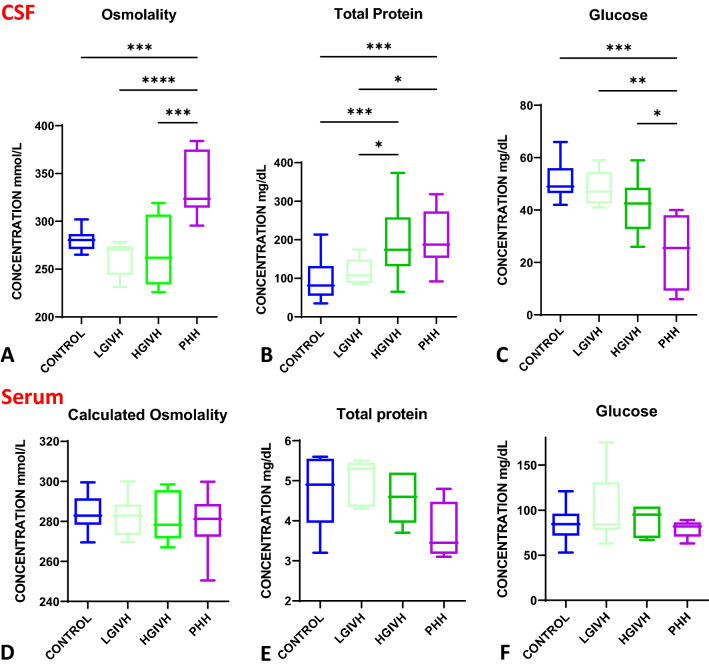
CSF and serum osmolality, total protein, and glucose in human infants with PHH. **A** chart showing increased CSF osmolality in PHH (n = 7) compared to control (n = 12), low grade IVH (n = 7), and high grade IVH (n = 7) (p = 0.0006, < 0.0001, 0.0003 respectively; Tukey’s post-test ANOVA). **B** chart showing increased total protein concentration in HGIVH (n = 11) and PHH (n = 11) compared to control (n = 16) (p = 0.0009 and 0.0006 respectively Tukey’s post-test ANOVA) and LGIVH (n = 9) (p = 0.034 and 0.028 respectively Tukey’s post-test ANOVA). **C** chart showing decreased glucose in PHH (n = 5) compared to control (n = 12), LGIVH (n = 7), and HGIVH (n = 7) (p = 0.0002, 0.0047, and 0.041 respectively Tukey’s post-test ANOVA). **D** chart showing no change in serum calculated osmolality across all the groups (ANOVA summary p-value = 0.71) (n = 19 control, 9 LGIVH, 7 HGIVH, and 11 PHH). **E** chart showing no change in serum total protein across all the groups (ANOVA summary p-value = 0.11) (n = 5 control, 5 LGIVH, 5 HGIVH, and 4 PHH). **F** chart showing no change in serum glucose across all the groups (ANOVA summary p-value = 0.37) (n = 12 control, 7 LGIVH, 7 HGIVH, 5 PHH)

CSF osmolality was elevated in PHH compared to all other groups. CSF osmolality in the control, LGIVH, HGIVH, and PHH group were 280.5 (15.6); 270.5 (30.0); 262.0 (73.0); and 323.5 (60.7) mmol/L, (median (Interquartile range [IQR])) respectively (Fig. 1). There was a 44.2 mmol/L absolute increase in mean osmolality in PHH CSF compared to control. Total protein was elevated in both HGIVH (median 174.3 mg/dL with IQR of 126.6) and PHH (median 187.5 mg/dL with IQR of 120.3) CSF relative to control (median 81.3 mg/dL with IQR of 76.9) and LGIVH (median 107.8 mg/dL with IQR of 61.5). CSF glucose was significantly lower in PHH (median 25.5 mg/dL with IQR of 28.7) compared to control (median 49.0 mg/dL with IQR of 9.5), LGIVH (median 47.0 mg/dL with IQR of 12), and HGIVH (median 42.5 mg/dL with IQR of 15.7). This represents a 27.5 mg/dL or 1.6 mmol/L decrease in the mean glucose concentration in PHH compared to control. There was no change in serum calculated osmolality, total protein, or glucose across all groups.

### CSF sodium, potassium, chloride

**Fig. 2:**
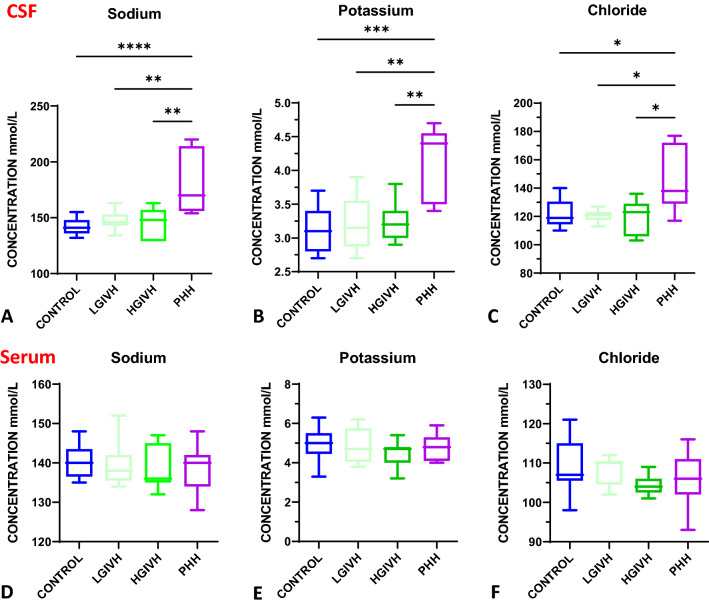
Increased Sodium, Potassium, and Chloride only in PHH. **A** chart showing increased CSF Na^+^ concentration in PHH (n = 7) compared to control (n = 12), low grade IVH (n = 7), and high grade IVH (n = 7) (p =  < 0.0001, 0.0017, 0.0013 respectively; Tukey’s post-test ANOVA). **B** chart showing increased CSF K^+^ concentration in PHH (n = 5) compared to control (n = 13), low grade IVH (n = 8), and high grade IVH (n = 7) (p = 0.0002, 0.0023, 0.0037 respectively; Tukey’s post-test ANOVA). **C** chart showing increased CSF Cl^−^ concentration in PHH (n = 7) compared to control (n = 13), low grade IVH (n = 6), and high grade IVH (n = 7) (p = 0.012, 0.023, 0.015 respectively; Tukey’s post-test ANOVA). **D** chart showing no change in serum sodium across all the groups (ANOVA summary p-value = 0.89) (n = 13 control, 9 LGIVH, 7 HGIVH, and 11 PHH). **E** chart showing no change in serum potassium across all the groups (ANOVA summary p-value = 0.57) (n = 13 control, 9 LGIVH, 7 HGIVH, and 11 PHH). **F** chart showing no change in serum chloride across all the groups (ANOVA summary p-value = 0.30) (n = 13 control, 9 LGIVH, 6 HGIVH, and 11 PHH)

CSF sodium (Na^+^) concentration was increased in PHH (median 170 mmol/L with IQR of 58) compared to all other groups (median (IQR): control 141.0 [12]; LGIVH 146.0 [10]; HGIVH 148.0 [28] mmol/L) (Fig. 2) consistent with a 38 mmol/L absolute increase in mean Na^+^ concentration in PHH compared to control. Similarly, CSF potassium (K^+^) concentration was elevated in PHH (median 4.4 mmol/L with IQR of 1.0) compared to control (median 3.1 mmol/L with IQR of 0.6), LGIVH (median 3.1 mmol/L with IQR of 0.7), and HGIVH (median 3.2 mmol/L with IQR of 0.4). Median and IQR for CSF chloride (Cl^−^) concentration in Control, LGIVH, HGIVH, and PHH were 119.0 (16.0); 121 (5.8); 123.0 (23.0); and 138.0 [43] mmol/L respectively. Mean CSF Cl^−^ concentration was elevated in PHH compared to control by 21 mmol/L. Notably, serum values for Na + , K + , and Cl- were similar across all groups.

### CSF magnesium, calcium, bicarbonate

CSF magnesium (Mg^2+^) concentration was elevated in PHH (3.7 ± 0.8 mg/dL) compared to control (2.6 ± 0.6 mg/dL; p = 0.031) and LGIVH (2.5 ± 0.3 mg/dL; p = 0.041) but not HGIVH (3.1 ± 0.6 mg/dL; p = 0.46) (Table 3). Concentration of calcium (Ca^2+^) was significantly higher in PHH (4.3 ± 0.3 mg/dL) compared to control (2.9 ± 0.7 mg/dL; p = 0.012), LGIVH (1.7 ± 0.8 mg/dL, p < 0.0001), but not HGIVH (3.3 ± 0.9 mg/dL, p = 0.21). This is a 1.0 mg/dL or 0.07 mmol/L absolute increase in mean Mg^2+^ concentration and 1.5 mmol/L increase in Ca^2+^ in PHH compared to control. Carbon dioxide (CO_2_) concentration, a reflection of bicarbonate ion concentration and pH, showed variable differences among groups with differences observed between PHH (19.8 ± 1.0 mmol/L) and HGIVH (19.5 ± 2.3 mmol/L) compared to LGIVH (13.3 ± 2.3 mmol/L; p = 0.019 and 0.010 respectively) but not control (17.3 ± 4.3 mmol/L). Similarly, there is no change in serum calcium or bicarbonate across all groups.

**Table 3:** Increased CSF Calcium and Magnesium in PHH but no difference in Bicarbonate compared to Control

		Control	LGIVH	HGIVH	PHH	P value
Magnesium	CSF	2.6 ± 0.6 (n = 13)	2.5 ± 0.3 (n = 6)	3.1 ± 0.6 (n = 6)	3.7 ± 0.8 (n = 4)	0.024 Control vs PHH (0.031); and LGIVH vs PHH (0.041)
Calcium	CSF	2.9 ± 0.7 (n = 15)	1.7 ± 0.8 (n = 7)	3.3 ± 0.9 (n = 7)	4.3 ± 0.3 (n = 4)	< 0.0001 Control vs LGIVH (0.013) and PHH (0.012); LGIVH vs HGIVH (0.0046) and PHH (< 0.0001)
	Serum	9.9 ± 0.5 (n = 9)	9.5 ± 0.6 (n = 5)	9.8 ± 0.4 (n = 6)	10.1 ± 0.3 (n = 5)	0.21
Bicarbonate	CSF	17.3 ± 4.3 (n = 13)	13.3 ± 2.3 (n = 7)	19.5 ± 2.3 (n = 6)	19.8 ± 1.0 (n = 4)	0.0062 LGIVH vs HGIVH (0.010) and PHH (0.019)
	Serum	24.9 ± 4.4 (n = 13)	25.1 ± 1.6 (n = 8)	24.3 ± 2.8 (n = 6)	25.9 ± 4.4 (n = 11)	0.85

Values recorded as mean ± standard deviation. In the “P value’ column, ANOVA summary p-value was recorded on the top row while significant pairwise post-test P value were recorded on the bottom row. All pairwise comparisons were analyzed based on Dunn’s or Tukey’s post-test ANOVA depending on the distribution of the data (normal distribution or not). n, represents sample size

### Degree of electrolytes change in CSF in PHH compared to control

The largest measured contributor to the osmolality change in PHH CSF appeared to be sodium (absolute change of 37.6 mmol/L), followed by chloride (20.9 mmol/L) (Table 4). Total protein contributed 5.9 mmol/L to the increase in osmolality in PHH CSF. However, it showed the largest change in concentration in the CSF, with an increase of 111.3% followed by glucose with a decrease of 55.2%.

**Table 4:** Absolute and Percent change in electrolyte concentrations in PHH compared to Control

	Control (mmol/L)	PHH (mmol/L)	Absolute change (mmol/L)	Percent change (%)
Total protein	5.3	11.2	5.9	111.3
Glucose	2.9	1.3	− 1.6	-55.2
Sodium	142.3	179.9	37.6	26.4
Potassium	3.1	4.1	1	32.3
Chloride	122.4	143.3	20.9	17.1
Bicarbonate	17.3	19.8	2.5	14.5
Calcium	0.16	0.24	0.08	50.0
Magnesium	0.14	0.21	0.07	50.0
Calculated Total Osmoles	296.2	364.1	67.9	
Measured Osmolality	280.8	336.5	55.7	19.8
Unaccounted Osmoles	15.4	27.6	12.2	

Absolute change is the difference in concentration between the Control and PHH values. Percent change is the difference divided by the control value and multiplied by 100. Calculated total osmoles is the sum of the electrolytes, total protein, and glucose in each group (control or PHH). Measured osmolality is the mean osmolality obtained from actual measurement with VAPRO osmometer. Unaccounted osmoles represent the difference in the calculated total osmoles and the measured osmolality

### Association between electrolytes, and osmolality and CSF cell counts

To further evaluate the possible causes of osmolality and electrolyte changes and its relationship to ventricular volume, we calculated the correlations shown in Additional file 1: Table S2. Osmolality positively correlated with Na (r,p = 0.55,0.0015), K (r,p = 0.51,0.0041), Cl (r,p = 0.60,0.0004), Ca (r,p = 0.49,0.0075), Mg (r,p = 0.58,0.0026) but not total protein (r,p = 0.11,0.59). Total nucleated cells positively correlated with osmolality, Na, K, Mg, and total protein (r,p = 0.48,0.025; 0.54,0.014; 0.49,0.024; 0.61,0.0036; 0.61,0.0008) but negatively correlated with glucose (r,p = -0.65, 0.0018). FOR was not correlated significantly with osmolality, Na, K, Cl or total protein (r,p = 0.48,0.11; 0.41,0.21; 0.48,0.12; 0.25,0.45; and 0.59,0.12). There were no significant correlations between individual electrolyte concentration, total protein, and osmolality with any individual WBC lineage (lymphocyte, neutrophil, monocyte, macrophage).

## Discussion

Osmolality and thus electrolyte concentration have been implicated in the development of ventriculomegaly [38, 54, 55]. However, no study has quantified CSF osmolality and electrolyte concentrations in humans with IVH/PHH. Our results show elevated CSF osmolality, total protein, Na, K, Cl, Mg, Ca in PHH compared to control. To further assess the relationship of the serum to the CSF, we evaluated these same electrolytes in the serum and observed no difference between the groups. With much evidence demonstrating PHH as a neuroinflammatory condition, we compared the WBC counts and differential in both CSF and serum. Our results showed total cells, total nucleated cells, and monocytes were higher in the CSF while only total WBC count was elevated in the serum with no changes in the cell differential in PHH compared to control. Finally, using correlation analyses, we evaluated the relationship of these parameters to ventricular size (using FOR) and cellularity (particularly total white blood cell and differential). We observed positive correlations between total nucleated cells and osmolality, sodium, and potassium. However, we observed no correlations between FOR and electrolytes concentration or osmolality.

Mean control CSF osmolality (280.8 mmol/L), Na (142.3 mmol/L), K (3.1 mmol/L), Cl (122.4 mmol/L), CO2 (17.3 mmol/L), were within the range of previously published studies [56]. Serum electrolyte concentration and osmolality were also within normal clinical reference range (Additional file 1: Table S1). The CSF osmolar gap (measured CSF osmolality difference from calculated CSF osmolality) (Table 4) could be due to unmeasured anions such as inorganic phosphates, and/or total free water content.

Osmolality is one of the driving forces for water movement across a cell layer [57, 58]. The contributors to osmolality are mostly ions but proteins also contribute to this osmotic pressure. Oncotic pressure is another driver of water transport and is created solely by protein concentration. The increase in CSF osmolality in PHH indicates an increase in the driving force of water into the ventricles and could be a major contributor to ventriculomegaly [38, 54, 55, 59]. Our results show that CSF osmolality in all IVH grades was similar to control but was elevated in PHH. It is interesting that clinically, IVH progresses to PHH when there is documented progressive ventricular dilatation and clinical signs of increased intracranial pressure (ICP). Normal electrolyte concentrations and osmolality in HGIVH compared to control even in the presence of ventricular enlargement (FOR > 0.5) [60] could indicate sufficient compensation by water transport into the CSF space. This compensation could maintain normal osmolality but be associated with larger ventricles, as observed in HGIVH. It is thus possible that, PHH is associated with uncompensated physiology with permissive increases in electrolyte and protein concentrations and osmolality, with progressive fluid imbalance, ventricular enlargement, and intracranial pressure [61] requiring CSF diversion.

The elevation in PHH CSF osmolality is interesting, as one would expect fluid movement to achieve equilibrium at or near normal CSF osmolality. Indeed, we found that PHH CSF osmolality was uniformly elevated relative to serum. This is likely due to limitation of water transport and thus ventricular dilatation by the ventricular wall compliance, hydrostatic pressure, and ICP [61]. In addition, the pathologic state (e.g. ChP hypersecretion, inflammation) in PHH may be sustained [15]; therefore, continuous electrolyte secretion without equivalent water transport into the ventricles leading to a dynamic equilibrium state. In this state, CSF osmolality might remain elevated even though the epithelium continues to transport water both transcellularly and paracellularly (if brain-CSF-barrier (BCSFB) is disrupted) [56] in an attempt to restore normal CSF osmolality. Another consideration is increased CSF osmolality should reasonably lead to cell shrinkage and activation of antidiuretic hormone (ADH); and some clinical observations show possible changes in ADH in hydrocephalus [62–66]. However, in a state of dynamic equilibrium and restriction to water transport into the ventricles by the factors mentioned earlier, it is possible for serum and epithelial cell osmolality to be normal while CSF osmolality remains elevated. Also, it is conceivable that if the BCSFB is disrupted in PHH as suggested by other studies [67, 68], water transport across the ChP will now become primarily paracellular (lower resistance flow) and this will reasonably exclude the cell as an intermediate compartment and cell osmolality and size might remain normal. At this point, the pathophysiology remains unclear, but our results suggest that there is aberrant transport of water across the different fluid compartments in PHH. As such, future studies to further evaluate total water content in the CSF and direction of water flow in PHH as well as ChP epithelial cell size and osmolality should be conducted. Recent studies have suggested that this water transport is likely disrupted in PHH [17, 69–71]. Other components of transport across the ChP epithelial (lateral intercellular space and/or claudin integrity and activity) should be investigated as well.

Water transport into the ventricular space could be originating from the ChP capillaries via AQP1 channels (if the BCSFB is intact) on the ChP apical membrane [72, 73] or from the brain parenchyma, sometimes mediated by AQP4 [74]. Unfortunately, AQP1 mediated water transport in PHH remains controversial. Some studies have shown decreased, unchanged, and increased activity of AQP1 in different forms of hydrocephalus [16, 17, 59, 75]. In the case of BCSFB disruption, which may occur in PHH, water transport across the ChP is no longer restricted to transcellular transport and may freely move down the osmotic gradient while being regulated by other forces such as hydrostatic pressure, ventricular wall compliance, and ICP as mentioned earlier. On the other hand, AQP4 is closely related to glymphatics, another putative system involved in CSF homeostasis. This system argues the existence of a fluid connection between the CSF space, brain parenchyma, and the perivascular spaces. Hydrostatic pressure generated by the arteries and AQP4 mediated water transport are two mechanisms by which the system moves fluid from the CSF space to the venous structures and peri-neural spaces [8, 71]. Impairment of the glymphatic clearance of CSF has been shown in IVH and PHH [71]. Serum and CSF osmolality do not vary much in normal state hence the osmotic pressure might not contribute much to this fluid movement in a “normal” infant. However, in a pathologic state such as PHH with such elevated CSF osmolality and reduced oncotic gradient (Additional file 1: Table S1), osmotic and oncotic pressures possibly become important drivers. The increased CSF osmotic and oncotic pressure in PHH may be one of the mechanisms, in conjunction with changes in AQP4 expression and distribution, which leads to the disruption of the glymphatic system described in IVH/PHH.

Similar to previous studies [34, 36, 37, 76], our results show that osmolality changes in the CSF are mostly driven by changes in electrolytes especially sodium and chloride concentration. As expected, sodium and chloride concentrations are elevated in PHH compared to control and all IVH grades. The change in sodium concentration contributes over 50% of the change in osmolality (Table 4). However, protein concentration is believed to contribute only minimally to osmolality [29, 37] and similarly, our results show that change in protein concentration only contributes about 8% to osmolality change (Table 4). In addition, compared to control, total protein concentrations are increased in both HGIVH and PHH but osmolality, sodium, and chloride concentrations are increased in PHH only. Osmolality also positively correlated with Na, K, Cl, Mg, and Ca but not total protein (Table 4). These all further support the notion that proteins, compared to electrolyte, likely contribute less to CSF osmolality changes.

PHH, though described clinically by signs and symptoms of ventriculomegaly and elevated ICP, is likely preceded by complex cytopathologies such as ChP CSF hypersecretion [15, 59, 77], inflammation [8, 77–79], and cell-junction disruption in the ventricular zone [11]. These pathologies are not restricted to PHH alone but also occur in IVH. Though the mechanism for development of PHH after IVH is unclear, these electrolyte changes are likely occurring after these cytopathologies as there are no observed changes in any IVH grade. Therefore, it is also unclear if these electrolyte changes are a cause or result of ventriculomegaly. It may appear they are at least likely contributors to ventricular dilatation [38, 54, 55] and development of PHH from IVH as explained earlier. Though the ependyma plays a significant role in water transport and CSF homeostasis; ion transport across it is not well described probably due to its highly permeable extracellular spaces [80] and the ChP being the major secretory organ in the central nervous system. The ChP is also one of the highest secretory epithelia in the body, contributing up to 80% of CSF production, and the major regulator of CSF content [72, 81] [72, 73, 82–84]. Thus, while the ChP epithelium is an important primary contributor to the electrolyte and osmolality changes we observed in this study, other possible contributors include BCSFB disruption and inflammatory processes. ChP hypersecretion occurs via increased expression of the NKCC1 [15, 59, 81], a bidirectional transporter of Na, K, and Cl involved in the net secretion of Cl into the ventricles for CSF production in normal state [56, 84]. NKCC1 channel hyperactivity might be responsible for increased CSF Na, K, and Cl ions in PHH; however, as described earlier, these changes would have to be in the context of abnormal water transport.

CO2, as a surrogate for bicarbonate which is the major driver of CSF pH [85], was not significantly increased compared to control; however, the CSF/serum ratio in PHH increased compared to control (Additional file 1: Table S1). It may be that our sample size was underpowered to detect differences between control and PHH. Any increased in CO2 might reflect activation of the Na dependent Cl transporter (NBCe2), which has been implicated in hydrocephalus [56, 59, 86]. It is important to note that CO2 passively diffuses across the BCSFB but normal serum CO2 and high CSF CO2 in PHH might suggest H^+^ and HCO3^−^ ion transport, implicating BCSFB disruption rather than CO2 diffusion. The sodium-hydrogen exchanger (NHE1), a channel that delivers sodium into the CSF while moving hydrogen ion out, might also be involved in CSF bicarbonate changes. If hyperactive in PHH, this could be contributing to the increased Na and pH in the CSF of infants with PHH. Further studies to examine the activity of both channels in PHH should be conducted.

Mg is also significantly increased in PHH compared to control, but it is unclear at this point what might be leading to this change. One possibility is the inflammatory response as Mg is positively correlated with nucleated cells. Total nucleated cells were also positively correlated with Na, K, osmolality, and total proteins indicating that inflammation might also be involved in the elevation of other CSF electrolytes and total protein. Many animals, in vitro, and human studies have shown increased cytokines and chemokines in PHH which might contribute to the total protein concentration [2, 18]. However, the increased total protein may be related to blood and its metabolites. Other studies [6, 29] have also shown increased total protein in PHH as well as increased hemoglobin, other blood metabolites, and hemoglobin associated proteins in PHH. Further emphasizing the possible involvement of inflammation in CSF biochemical profile changes is decreased glucose in PHH compared to control, LGIVH, and HGIVH. Decreased CSF glucose is clinically associated with neuro-inflammation as it is a common finding across many neuro-inflammatory conditions including meningitis. Altogether, these solutes (total protein, Mg, Ca, glucose) likely have minor contribution to osmolality changes.

The disruption of the BCSFB is another pathology that might contribute to the electrolyte, total protein, and osmolality changes [33, 59, 87, 88]. Of note, CSF osmolality and the concentrations of many electrolytes exceed serum levels in PHH. This suggests that the electrolyte changes were not simply the result of serum and CSF mixing. In addition, the serum concentration of all electrolytes did not change in PHH compared to control. On the other hand, impairment in macromolecule efflux channels present on the BCSFB have been described in hydrocephalus [55, 89]. These changes were associated with CSF osmolality and might also contribute to the complex etiology of CSF hyperosmolality in PHH. Our data indicate that CSF electrolyte and osmolality changes are due to more complex processes, including ChP hypersecretion or inflammation.

Finally, maintaining a normal CSF biochemical profile is essential for normal neurodevelopment and neuronal activity especially at the neonatal age period [90]. CSF contents have been shown to affect neural progenitor cell behavior (proliferation and differentiation), brain patterning, and neurogenesis [87, 91, 92] and may be related to some of the neurodevelopmental impairments observed in children with PHH. This further emphasizes the importance of this study and future studies should evaluate the interaction of CSF electrolytes, the neurogenic niche, and neurodevelopmental outcomes.

## Limitations

A few limitations in this study must be acknowledged. IVH/PHH is more prevalent in males with about 60% of cases affecting males [93–95] but, of note, our cohort includes a higher percentage of males (about 77%) and thus could introduce bias in the results. However, in our subgroup analyses to examine the effect of sex on biochemical profile, we did not observe differences (Additional file 1: Tables S3–S4). Sample variation (including high standard deviations in some analyses) due to limited sample size and inevitable variation in sample acquisition, processing, storage, as well as natural clinical heterogeneity within and between groups. Though we are unable to determine the precise time germinal matrix hemorrhage occurred, about 50% of IVH cases occur within 6–8 h after birth and 90% by 3 days after birth [96, 97]. The time from birth to LP for control, LGIVH, HGIVH, PHH are 7.1 ± 4.8, 1.5 ± 1.8, 4.7 ± 3.8, and 2.5 ± 1.3 weeks respectively. The LGIVH group had earlier LP sampling compared to the control only (p = 0.001). LPs were obtained for clinical purposes per the primary care team accounting for the variability in timing of sample acquisition. Lumbar CSF samples were used in this study, but ventricular CSF samples will be evaluated in future studies to address possible rostral-caudal gradient differences. This study utilized a small sample size especially in some of the electrolyte analysis and future studies with larger sample size need to be conducted to further validate the results of this study.

## Conclusions

In this study, we report the novel finding of increased CSF concentration of Na, K, Cl, Ca, Mg, and osmolality in infants with PHH. Changes in CSF osmolality are most likely driven by changes in sodium and electrolyte concentrations rather than protein concentrations. These changes in the CSF were independent of the serum as we found no changes in serum electrolytes across the groups. However, we observed positive correlations between CSF total nucleated cells and CSF osmolality, Na, and K. Total cells and nucleated cells in the CSF were increased in PHH. Serum WBC count was also increased in PHH but no differences in the WBC differential. These findings provide important insights into the pathophysiology of IVH and PHH.

## Supplementary Information

**Additional file 1: Table S1**. CSF and serum electrolyte concentrations in control individuals as well as CSF/Serum electrolyte ratio in control and PHH. **Table S2.** Correlations between electrolytes and osmolality, and CSF cell counts. **Table S3**. Subgroup analysis by sex of cerebrospinal fluid total protein, osmolality, and electrolytes in PHH. Sample size limitations permitted analysis only of the male cohort; there were insufficient samples for analysis by female sex. To address this limitation, regression analysis examining sex as an explanatory variable was also performed (please refer to Table S4). Values recorded as mean ± standard deviation. In the “P value’ column, ANOVA summary p-value was recorded on the top row while significant pairwise post-test P value were recorded on the bottom row. All pairwise comparisons were analyzed based on Dunn’s or Tukey’s post-test ANOVA depending on the distribution of the data (normal distribution or not). n, represents sample size. **Table S4**. F, t, and p values from regression analyses evaluating sex as an explanatory variable. Analysis of all CSF parameters resulted in non-significant p-values indicating sex is not an explanatory variable for differences in the CSF parameters. F=F-value; t=t-value; and p=p-value. **Figure S1.** Ultrasonographic image representation of Control, IVH and PHH.

## Data Availability

The datasets used and/or analyzed during the current study are available from the corresponding author on reasonable request.
