# Supplementary material for: Biochemical profile of human infant cerebrospinal fluid in intraventricular hemorrhage and post-hemorrhagic hydrocephalus of prematurity
Source: Fluids Barriers CNS. 2021 Dec 24;18:62. doi: 10.1186/s12987-021-00295-8 (PMC8710025; doi:10.1186/s12987-021-00295-8)
Supplement: Supplementary file 1 — Additional file 1: Table S1. CSF and serum electrolyte concentrations in control individuals as well as CSF/Serum electrolyte ratio in control and PHH. Table S2. Correlations between electrolytes and osmolality, and CSF cell counts. Table S3. Subgroup analysis by sex of cerebrospinal fluid total protein, osmolality, and electrolytes in PHH. Sample size limitations permitted analysis only of the male cohort; there were insufficient samples for analysis by female sex. To address this limitation, regression analysis examining sex as an explanatory variable was also performed (please refer to Table S4). Values recorded as mean ± standard deviation. In the “P value’ column, ANOVA summary p-value was recorded on the top row while significant pairwise post-test P value were recorded on the bottom row. All pairwise comparisons were analyzed based on Dunn’s or Tukey’s post-test ANOVA depending on the distribution of the data (normal distribution or not). n, represents sample size. Table S4. F, t, and p values from regression analyses evaluating sex as an explanatory variable. Analysis of all CSF parameters resulted in non-significant p-values indicating sex is not an explanatory variable for differences in the CSF parameters. F=F-value; t=t-value; and p=p-value. Figure S1. Ultrasonographic image representation of Control, IVH and PHH. [file 12987_2021_295_MOESM1_ESM.docx]

**Supplementary Data**

**Artificial CSF Recipe**

1. Glucose 1440 mg, KCl 178.9 mg, KH2PO4 136 mg, MgCl2 152.6 mg, CaCl2 235.2 mg into separate tube

2. Add 14 ml of H2O into each tube, this is group 1

3. NaCl 5798 mg, NaHCO3 1746 mg into separate tubes and add 28 ml of H2O into these tubes; this is group 2

4. Add 30ml H2O into a separate tube called “final” tube

5. Add 0.7 ml solution from each tube in Group 1 and 1.4 ml solution from each tube in Group 2 into the “final” tube

6. Add H2O up to 40 ml total volume.

aCSF samples were diluted to achieve electrolyte concentrations needed to determine linearity during the validation process.

**Comparison between Serum and Electrolyte Concentrations in Control and PHH**

Consistent with previously published data (1-3), CSF concentration of chloride and bicarbonate are higher than serum levels in control infants. Serum levels of total protein, glucose, potassium, and calcium are higher than CSF levels in control infants. As expected, CSF/Serum ratio of osmolality, total protein, sodium, potassium, chloride, calcium were elevated in PHH compared to control while CSF/Serum ratio of glucose was lower.

|  | Serum | CSF | P value | CSF/Serum ratio (Control) | CSF/Serum ratio (PHH) |
| --- | --- | --- | --- | --- | --- |
| Osmolality (mmol/L) | 284.3 ± 8.6 | 280.8 ± 11.0 | 0.38 | 0.99 | 1.20 |
| Total Protein (mg/dL) | 4780 ± 962.8 | 94.8 ± 47.5 | <0.000001***** | 0.020 | 0.055 |
| Glucose (mg/dL) | 85.5 ± 17.9 | 51.8 ± 7.5 | 0.000002***** | 0.61 | 0.31 |
| Sodium (mmol/L) | 140.3 ± 4.3 | 142.3 ± 7.1 | 0.41 | 1.01 | 1.30 |
| Potassium (mmol/L) | 5.0 ± 0.8 | 3.1 ± 0.3 | <0.000001***** | 0.62 | 0.85 |
| Chloride (mmol/L) | 109.2 ± 6.7 | 122.4 ± 9.7 | 0.00051*** | 1.12 | 1.35 |
| Bicarbonate (mmol/L) | 24.9 ± 4.3 | 17.3 ± 4.3 | 0.00015*** | 0.69 | 0.76 |
| Calcium (mg/dL) | 9.9 ± 0.5 | 3.0 ± 0.8 | <0.000001***** | 0.30 | 0.42 |
| Magnesium (mg/dL) | 2.3 ± 0.3 | 2.6 ± 0.6 | 0.37 | 1.13 | --- |

**Supplementary Table 1:** CSF and serum electrolyte concentrations in control individuals as well as CSF/Serum electrolyte ratio in control and PHH

Values are recorded as Mean ± standard deviation. P value obtained from t-test analysis based on distribution (normal distribution or not). Table shows decreased concentration of total protein, glucose, potassium, and calcium in CSF compared to serum but an increased concentration of chloride and bicarbonate in CSF compared to serum.

* represents degree of significance

**Correlations between CSF Osmolality, Electrolyte, FOR, and Cell Counts**

|  | Osmolality | FOR | Total Cells | Nucleated Cells | Lymphocytes | Neutrophils | Monocytes | Macrophages |
| --- | --- | --- | --- | --- | --- | --- | --- | --- |
| Osmolality | --- | --- | 0.35 (0.098) | 0.48* (0.025) | 0.23 (0.21) | 0.30 (0.22) | -0.18 (0.38) | -0.58 (0.088) |
| Total Protein | 0.11 (0.59) | 0.59 (0.12) | 0.41 (0.023) | 0.61*** (0.0008) | -0.35 (0.067) | 0.35 (0.11) | -0.35 (0.070) | 0.25 (0.52) |
| Sodium | 0.55** (0.0015) | 0.41 (0.21) | 0.38 (0.094) | 0.54* (0.014) | -0.080 (0.72) | 0.45 (0.092) | -0.31 (0.14) | 0.0084 (0.99) |
| Potassium | 0.51** (0.0041) | 0.48 (0.12) | 0.18 (0.44) | 0.49* (0.024) | 0.11 (0.62) | 0.37 (0.15) | -0.28 (0.18) | -0.32 (0.44) |
| Chloride | 0.60*** (0.0004) | 0.25 (0.45) | 0.33 (0.13) | 0.35 (0.11) | 0.014 (0.95) | 0.29 (0.25) | -0.093 (0.66) | 0.17 (0.67) |
| Bicarbonate | 0.20 (0.32) | 0.48 (0.098) | 0.23 (0.31) | 0.074 (0.76) | -0.061 (0.79) | 0.37 (0.18) | -0.43* (0.040) | 0.34 (0.40) |
| Calcium | 0.49** (0.0075) | 0.62* (0.025) | 0.37 (0.071) | 0.32 (0.15) | -0.065 (0.76) | 0.40 (0.12) | -0.26 (0.21) | -0.094 (0.82) |
| Magnesium | 0.58** (0.0026) | 0.46 (0.15) | 0.57** (0.0052) | 0.61** (0.0036) | -0.27 (0.22) | 0.32 (0.27) | -0.058 (0.79) | -0.039 (0.93) |
| Glucose | -0.30 (0.13) | -0.87* (0.024) | -0.48* (0.025) | -0.65** (0.0018) | -0.18 (0.42) | -0.46 (0.096) | 0.41 (0.053) | -0.36 (0.39) |
| FOR | 0.48 (0.11) | --- | 0.67 (0.27) | 0.63 (0.50) | 0.12 (0.84) | 0.12 (0.84) | -0.46 (0.37) | 0.00 (>0.99) |

**Supplemental Table 2:** Correlations between electrolytes and osmolality, and CSF cell counts

Values are recorded as correlation coefficient (p-value). Osmolality is correlated with sodium, potassium, chloride, calcium, and magnesium. Total nucleated cells is correlated with osmolality, sodium, potassium, magnesium, glucose, and total protein. Cell differentials are recorded as percentage of total WBC. Total cells and nucleated cells are recorded as density in cells/mcl. Spearman or Pearson correlations are used depending on if data is normally distributed.

* represents degree of significance.

**CSF Profile Analysis to Account for Variability due to Sex**

In order to control for the potential influence of infant sex on CSF parameters given the high percentage of male samples in the study cohort, we conducted a subgroup analysis on only male samples. Notably, the number of female samples was not sufficient for a similar subgroup analysis on female only samples. The results of the analysis on male samples showed that osmolality, sodium, and potassium were elevated in PHH compared to control (P=0.0045, 0.016, and 0.0099 respectively Dunn Post-test ANOVA) while total protein was elevated in both HGIVH, and PHH compared to control (p=0.0042 and 0.0026 respectively Dunn Post-test ANOVA). CSF chloride was elevated in PHH compared to HGIVH (p=0.032) while calcium was elevated in HGIVH, and PHH compared to LGIVH (p=0.035 and 0.0038 respectively). CSF glucose showed difference across the group (p=0.037 ANOVA summary) but no differences on post-test multiple comparisons using Dunn’s test. Finally, CSF bicarbonate and magnesium showed no differences between the groups (p=0.075, and 0.07 respectively ANOVA summary).

|  | Control | LGIVH | HGIVH | PHH | P value |
| --- | --- | --- | --- | --- | --- |
| Osmolality (mmol/L) | 279.8 ± 9.4 (n=8) | 273.9 ± 3.1 (n=4) | 270.9 ± 36.4 (n=7) | 339.1 ± 37.4 (n=5) | 0.0013 |
|  |  |  |  |  | PHH vs Control (0.0045), LGIVH (0.0080), and HGIVH (0.0016) |
| Sodium (mmol/L) | 147.7 ± 16.3 (n=9) | 149.0 ± 7.8 (n=6) | 146.1 ± 13.3 (n=7) | 181.5 ± 28.8 (n=6) | 0.02 |
|  |  |  |  |  | Control vs PHH (0.016) |
| Potassium (mmol/L) | 3.1 ± 0.7 (n=11) | 3.8 ± 0.3 (n=6) | 3.2 ± 0.3 (n=7) | 4.1 ± 0.6 (n=5) | 0.016 |
|  |  |  |  |  | Control vs PHH (0.0099) |
| Chloride (mmol/L) | 125.5 ± 14.2 (n=10) | 122.0 ± 3.1 (n=5) | 120.1 ± 12.2 (n=7) | 145.3 ± 24.0 (n=6) | 0.03 |
|  |  |  |  |  | HGIVH vs PHH (0.032) |
| Glucose (mg/dL) | 53.0 ± 8.2 (n=10) | 65.5 ± 35.9 (n=4) | 38.2 ± 7.9 (n=5) | 30.3 ± 10.6 (n=3) | 0.037 |
|  |  |  |  |  |  |
| Calcium (mmol/L) | 3.0 ± 0.6 (n=10) | 1.6 ± 0.5 (n=5) | 3.3 ± 0.9 (n=6) | 4.3 ± 0.4 (n=3) | 0.0036 |
|  |  |  |  |  | LGIVH vs HGIVH (0.035) and PHH (0.0038) |
| Magnesium (mmol/L) | 2.5 ± 0.6 (n=9) | 2.6 ± 0.4 (n=4) | 3.1 ± 0.6 (n=6) | 3.6 ± 1.0 (n=3) | 0.07 |
| Bicarbonate (mmol/L) | 17.1 ± 5.7 (n=11) | 13.8 ± 2.5 (n=5) | 19.5 ± 2.3 (n=6) | 19.3 ± 0.6 (n=3) | 0.075 |
| Total protein (mg/dL) | 102.0 ± 50.5 (n=11) | 124.8 ± 35.8 (n=6) | 218.0 ± 76.8 (n=9) | 218.9 ± 66.9 (n=7) | 0.0004 |
|  |  |  |  |  | Control vs HGIVH (0.0042) and PHH (0.0026) |

**Supplementary Table 3:** Subgroup analysis by sex of cerebrospinal fluid total protein, osmolality, and electrolytes in PHH. Sample size limitations permitted analysis only of the male cohort; there were insufficient samples for analysis by female sex. To address this limitation, regression analysis examining sex as an explanatory variable was also performed (please refer to Supplementary Table 4). Values recorded as mean ± standard deviation. In the “P value’ column, ANOVA summary p-value was recorded on the top row while significant pairwise post-test P value were recorded on the bottom row. All pairwise comparisons were analyzed based on Dunn’s or Tukey’s post-test ANOVA depending on the distribution of the data (normal distribution or not). n, represents sample size.

Finally, we also conducted regression analyses to evaluate if sex is an explanatory variable for the observed differences in CSF osmolality, total protein, and electrolytes. The results and found that sex is not a significant explanatory variable for the differences observed across the groups in all CSF parameters measured.

|  | Osmolality | Total Protein | Sodium | Potassium | Chloride | Bicarbonate | Glucose | Magnesium | Calcium |
| --- | --- | --- | --- | --- | --- | --- | --- | --- | --- |
| F | 0.36 | 1.86 | 0.85 | 1.69 | 0.35 | 0 | 4.11 | 0.16 | 0.89 |
| t | 0.6 | 1.36 | 0.92 | 1.3 | 0.59 | -0.02 | 2.03 | 0.4 | -0.94 |
| p | 0.56 | 0.18 | 0.36 | 0.2 | 0.56 | 0.98 | 0.055 | 0.69 | 0.36 |

**Supplementary Table 4:** F, t, and p values from regression analyses evaluating sex as an explanatory variable. Analysis of all CSF parameters resulted in non-significant p-values indicating sex is not an explanatory variable for differences in the CSF parameters. F=F-value; t=t-value; and p=p-value.


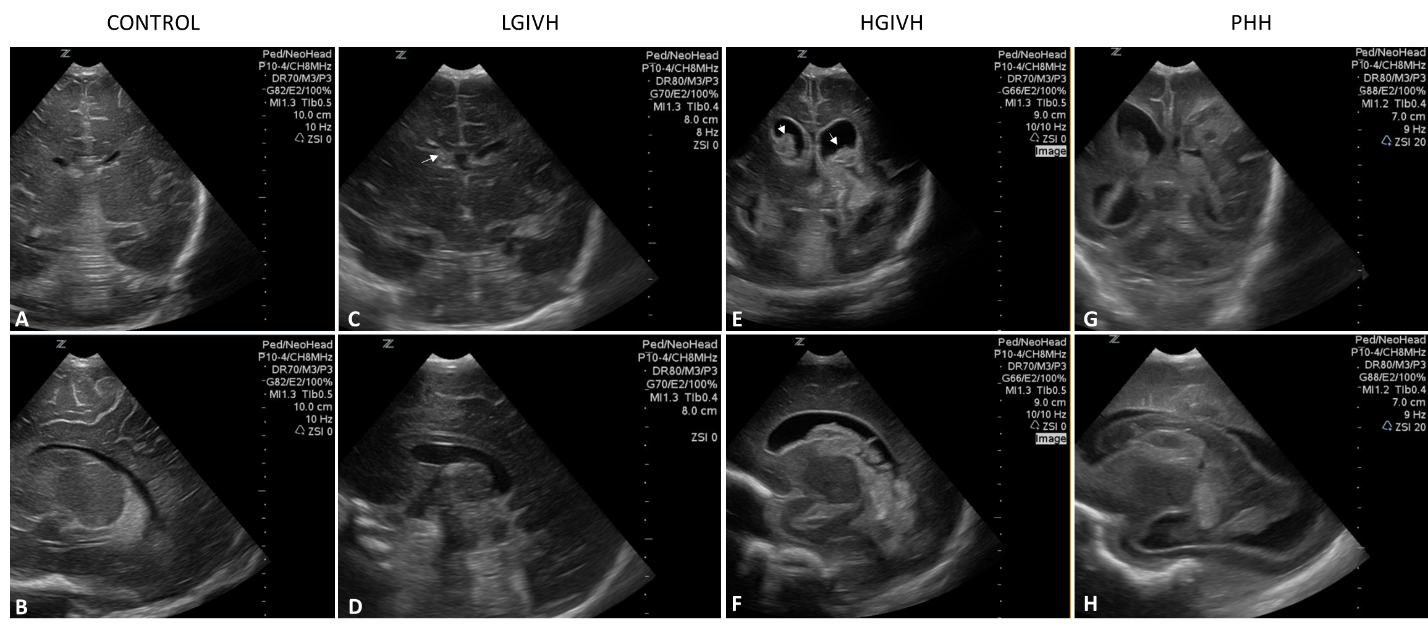


**Supplementary Figure 1**: Ultrasonographic image representation of Control, IVH and PHH

**A,B;** Coronal and sagittal images, respectively, of a control case showing normal ventricular size with no intracranial hemorrhage. **C,D;** Coronal and sagittal images, respectively, of a case with LGIVH showing right greater than left subependymal hemorrhages (arrow head denotes right subependymal hemorrhage) with no ventriculomegaly, intraparenchymal hemorrhage or periventricular leukomalacia. **E,F;** Coronal and sagittal images, respectively, of a HGIVH case showing bilateral ventriculomegaly with intra ventricular hemorrhage (arrow head denotes subependymal hemorrhages). **G,H;** Coronal and sagittal images, respectively, of a PHH case showing bilateral ventriculomegaly with left-sided intraparenchymal hemorrhage. Aggregate ventricular size denoted by frontal-occipital horn ratio (FOR) for each group (control, LGIVH, HGIVH, PHH) are recorded in table 1.

1. Akaishi T, Takahashi T, Nakashima I, Abe M, Aoki M, Ishii T. Osmotic pressure of serum and cerebrospinal fluid in patients with suspected neurological conditions. Neural regeneration research. 2020;15(5):944.

2. Damkier HH, Brown PD, Praetorius J. Cerebrospinal fluid secretion by the choroid plexus. Physiological reviews. 2013;93(4):1847-92.

3. Praetorius J, Damkier HH. Transport across the choroid plexus epithelium. American Journal of Physiology-Cell Physiology. 2017;312(6):C673-C86.
